# Detection and differentiation of bacterial spores in a mineral matrix by Fourier transform infrared spectroscopy (FTIR) and chemometrical data treatment

**DOI:** 10.1186/2046-1682-4-14

**Published:** 2011-07-14

**Authors:** Andrea Brandes Ammann, Helmut Brandl

**Affiliations:** 1 University of Zurich, Institute of Evolutionary Biology and Environmental Studies, Winterthurerstrasse 190, CH-8057 Zurich, Switzerland

**Keywords:** *Bacillus *spores, infrared spectroscopy, FTIR, clay minerals, bentonite, mineral matrix

## Abstract

**Background:**

Fourier transform infrared spectroscopy (FTIR) has been used as analytical tool in chemistry for many years. In addition, FTIR can also be applied as a rapid and non-invasive method to detect and identify microorganisms. The specific and fingerprint-like spectra allow - under optimal conditions - discrimination down to the species level. The aim of this study was to develop a fast and reproducible non-molecular method to differentiate pure samples of *Bacillus *spores originating from different species as well as to identify spores in a simple matrix, such as the clay mineral, bentonite.

**Results:**

We investigated spores from pure cultures of seven different *Bacillus *species by FTIR in reflection or transmission mode followed by chemometrical data treatment. All species investigated (*B. atrophaeus, B. brevis, B. circulans, B. lentus, B. megaterium, B. subtilis, B. thuringiensis*) are typical aerobic soil-borne spore formers. Additionally, a solid matrix (bentonite) and mixtures of benonite with spores of *B. megaterium *at various wt/wt ratios were included in the study. Both hierarchical cluster analysis and principal component analysis of the spectra along with multidimensional scaling allowed the discrimination of different species and spore-matrix-mixtures.

**Conclusions:**

Our results show that FTIR spectroscopy is a fast method for species-level discrimination of *Bacillus *spores. Spores were still detectable in the presence of the clay mineral bentonite. Even a tenfold excess of bentonite (corresponding to 2.1 × 10^10 ^colony forming units per gram of mineral matrix) still resulted in an unambiguous identification of *B. megaterium *spores.

## Background

Infrared spectroscopy of a chemical compound or material is based on the absorption of radiation energy in the infrared range (near infrared, NIR, 780 nm to 2.5 μm, 12821 to 4000 cm^-1^; mid infrared, MIR, 2.5 to 25 μm, 4000 to 400 cm^-1^; far infrared FIR, 25 μm to 1 mm, 400 to 10 cm^-1^). Absorption peaks correspond to vibrations or rotations of the chemical bonds (e.g., C-H, N-H, or C-O) between the atoms making up the material. Because each different material is a unique combination of atoms, no two compounds produce exactly the same infrared spectrum resulting in a characteristic infrared "fingerprint". Consequently, infrared spectroscopy can result in a positive identification of basically every different kind of material. Although infrared spectroscopy has been routinely used as analytical tool in chemistry for many years, this technique has only recently been applied in environmental microbiology or microbial ecology.

In 1911, W.W. Coblentz (cited by [1]) was the first to analyze complex biological samples such as gelatine or chitin as well as minerals (e.g., quartz, opal, and muscovite) by infrared spectroscopy. The first infrared spectroscopy analyses in biological experiments were conducted in the early fifties. Most of them were on a more general level (e.g., [2-5]) and only a few were applied in medical diagnostics or in food production [6-8]. At that time, the instruments and possibilities for subsequent data processing were much more limited than today. In the eighties, Naumann and coworkers [9] developed efficient methods and instruments for microbiological use in a joint project between the Robert Koch Institute and a manufacturer of FTIR spectrometers (Bruker, Germany).

Fourier transform infrared (FTIR) spectroscopy in the MIR range can be applied as a rapid and non-invasive physico-chemical method to detect and identify microorganisms [9]. More recently, methods - particularly statistical data treatments - have been further developed to get faster and better results. Whole living cells can be analyzed non-destructively, which allows *in vivo *investigations. As example, diffusive reflectance infrared spectroscopy (DRIFT) was used to discriminate among 36 strains of vegetative *Bacillus *cells and their spores [10]. More recently, different serovars of *Salmonella enterica *have been discriminated by mid-FTIR in attenuated total reflection (ATR) mode applying soft independent modeling of class analogy (SIMCA modelling) [11]. Discrimination of endospores by mid-FTIR in ATR mode followed by the application of principal component analysis (PCA), hierarchical cluster analysis (HCA), and SIMCA remained possible even after autoclaving of the samples [12]. Libraries have been developed to relate spectral absorbance peaks of key functional groups present in proteins, carbohydrates, lipids, or nucleic acids [13]. Spectra of biological samples can be divided in different regions or windows. The typical fingerprint region for microorganisms is between wavenumbers of 650 cm^-1 ^and 1800 cm^-1 ^originating from cellular carbohydrate compounds and proteins. Cellular fatty acids and lipids show peaks between 2800 and 3000 cm^-1^. Best classifications are obtained using spectral differences in the amide I and II regions of 1500 to 1650 cm^-1 ^[14]. Basic principles and applications in biology are described in detail by Naumann [15]. A short summary of publications concerning the discrimination, classification, and identification of microorganisms as whole cells is given by [16]. Our aims were (i) to discriminate spores of different soil-borne bacilli; and (ii) to detect bacterial spores in the presence of a clay mineral matrix serving as a simple surrogate for soil. Most research has been done so far on pure bacterial cultures in the absence of matrices, for example in studies related to medicine, diagnostics, or food production [17].

## Results and Discussion

FTIR is a rapid and easy method to differentiate microorganisms down to the molecular level without the need of a complex and often time-consuming sample preparation. For measurement in the ATR mode (reflection), solid samples can be placed directly on the crystal prism of the ATR accessory. Transmission mode requires suspension for solids and always a drying step before the measurement. Most of the studies done so far used the transmission method. Nevertheless, Baldauf and coworkers found ATR to give better resolution of peaks while using less biomass compared to other FTIR methods [18].

**Figure 1:**
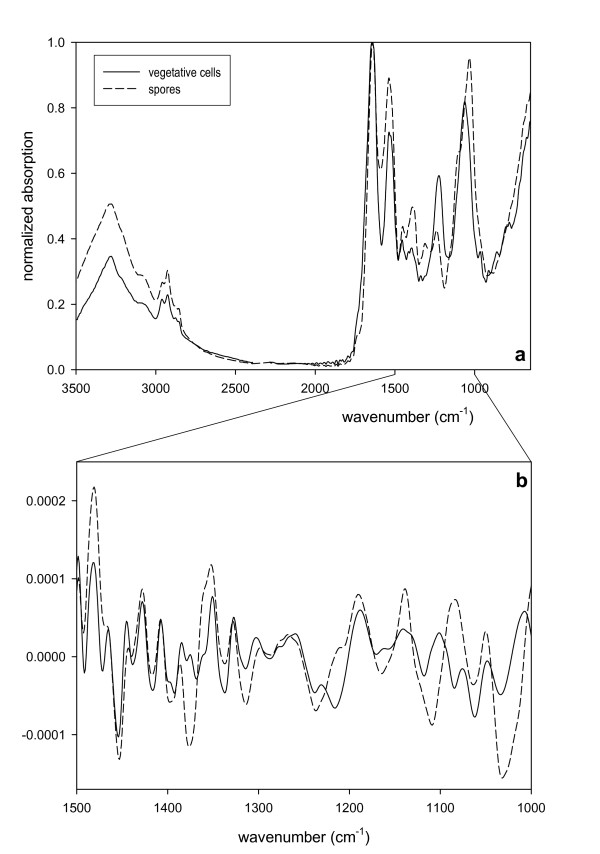
**FTIR spectra of vegetative cells of *Bacillus circulans *(solid line) and its spores (dashed line), measured in ATR mode (ZnSe prism)**. a) normalized spectra; b) second derivative of spectral range 1000 to 1500 cm^-1^.

**Figure 2:**
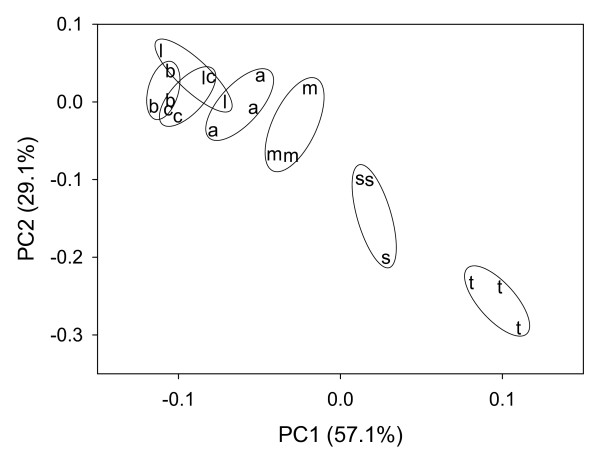
**Differentiation by principal component analysis of spores formed by seven *Bacillus *species measured in FTIR-ATR mode**. a: *B. atrophaeus*; b: *B. brevis*; c: *B. circulans*; m: *B. megaterium*; l: *B. lentus*; s: *B. subtilis*; t: *B. thuringiensis*. Measurements were performed in triplicates.

*Bacillus *species used in this study (such as *B. atrophaeus, B. brevis, B. circulans, B. lentus, B. megaterium, B. subtilis*, and *B. thuringiensis*) are typical representatives of aerobic soil-borne spore formers and have been isolated from various soil environments. As an example, *B. subtilis *is a typical member of spore forming bacilli. In some cases, *B. megaterium *might occur in soil at high densities [19-21]. Usually, a discrimination of *Bacillus *spores by classical (e.g., microscopical) methods is rather difficult and tedious [22]. Discrimination of vegetative cells and spores of *Bacillus circulans *was possible using FTIR and subsequent chemometrical analysis of the spectra (Figure 1). In particular, original spectra and second derivatives thereof showed distinct inter-specific differences between 1000 and 1500 cm^-1^. Differential spectra of second derivatives showed the most significant differences in spectral ranges of 1025 to 1032, 1374 to 1376, 1440 to1455, 1616 to 1619, and 1633 to 1644 cm^-1^. Most important, even spores of different *Bacillus *species can be distinguished by chemometrical data treatment using PCA (Figure 2). By FTIR spectroscopy, spores of *Bacillus thuringiensis, B. subtilis*, and *B. megaterium *were easily distinguished. Our data are in good agreement with results of other investigations [23]. In some cases, however, IR fingerprints obtained by chemometrical analysis of spores of *B. atrophaeus, B. brevis, B. circulans, and B. lentus *clustered close together making a discrimination difficult (Figure 2).

**Figure 3:**
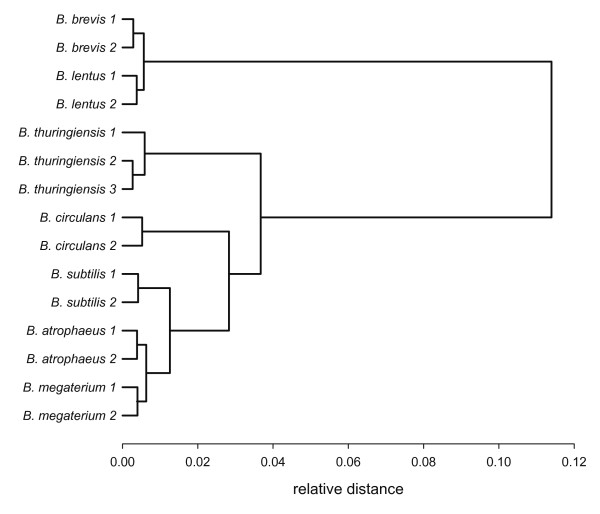
**Differentiation of spores from seven *Bacillus *species measured in FTIR-transmission mode on PE film by hierarchical cluster analysis**. Measurements were performed in replicates indicated by numbers.

When we used hierarchical cluster analysis (HCA) instead of PCA for the discrimination of spores, similar results were obtained and differentiation was possible (Figure 3). However, slight differences were observed between the statistical treatments, probably due to the different FTIR mode applied, namely transmission instead of ATR. Remarkably, the reclassification of *Bacillus brevis *as *Brevibacillus brevis *based on molecular markers [24] is reflected in early branching of the hierarchical clustering tree. Moreover, the distance trees resulting from hierarchical cluster analysis based on FTIR investigations of pure cultures were in agreement to phylogenetic trees derived from classical molecular methods based on 16S rRNA gene sequences (see e.g., [25]) and supports findings of other studies [26,27].

**Figure 4:**
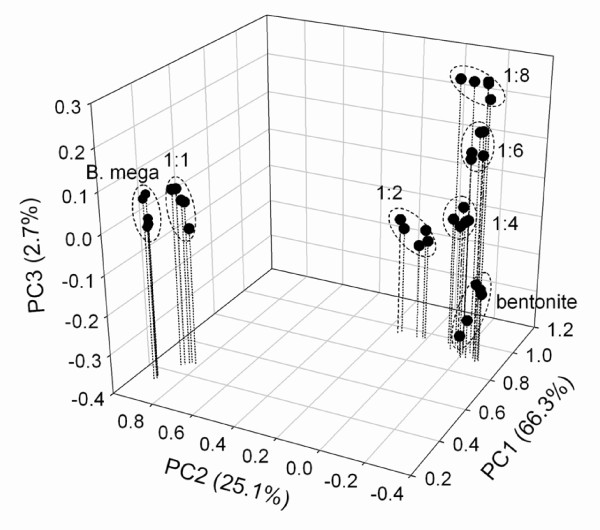
**Multiscaling principal component analysis (PCA) of spores of *Bacillus megaterium *in the presence of the clay mineral bentonite at different spore/bentonite (wt/wt) ratios**. "B mega": *Bacillus megaterium *without bentonite; "bentonite": pure bentonite without spores. Second derivatives of original spectra were truncated (750 - 1800 cm^-1^) and used for PCA. Five independent replicates per sample were measured. Drop lines are given for the PC1/PC2 plane.

To test our ability to discriminate spores in the presence of a matrix, the ATR mode was applied to study mixtures of pure spores and a clay mineral (bentonite). *Bacillus *spores (as well as vegetative cells) usually adhere strongly to different solid surfaces including clay minerals such as kaolinite or bentonite [28,29]. The latter might be used as simple matrix and a surrogate for soil. Generally, silicate minerals (such as e.g., bentonite) strongly absorb IR radiation, in particular in the 950 to 1100 cm^-1 ^region due to Si-O bond stretching, resulting in a typical IR spectrum [30,31]. This strong background absorption makes the determination of second phases in the matrix (e.g., bacterial spores) rather difficult. Differential spectra of second derivatives showed most prominent differences in the spectral range of 1652 to 1655 cm^-1^, attributable to amide I vibrational bands of proteins [13]. Our investigations show that it is possible to differentiate spore-bentonite mixtures at different mixing ratios by using multiscaling principal component analysis of second derivatives of FTIR spectra (Figure 4). In particular, bentonite samples with a high spore content (1:1 wt/wt) were easily discriminated from others. Even an approximately tenfold excess of bentonite (1:8 wt/wt ratio) still resulted in a differentiation from pure bentonite indicating the presence of *Bacillus *spores. In terms of colony forming units (cfu), a 1:8 spores/bentonite ratio (wt/wt) corresponded to 2.1 × 10^10 ^cfu per gram. In an earlier investigation, we investigated the occurrence of bacterial endospores in soils from various locations including grasslands (pasture, meadow), allotment gardens, and forests, as well as fluvial sediments, using a method based on the fluorescence of terbium [32]. Spore counts in the range of up to 10^9 ^spores per gram of dry soil were found in grassland soils. Foster et al. [33] performed similar FTIR experiments including clay minerals with different *Bacillus *species (vegetative cells and spores) and obtained comparable results.

## Conclusions

Our study shows that spores originating from different *Bacillus *species can be discriminated by applying FTIR and subsequent multiscaling chemometrical data treatment. Additionally - and more important - spores were still detectable in the presence of clay mineral matrix such bentonite which was used as a simple surrogate of soil. An important advantage of FTIR is the small amount of sample to be used. Only a few milligrams are needed to perform a measurement. In principle, data processing and identification of spores or vegetative cells can be automated. There are fully automated devices available in other fields to identify chemical substances, especially when rapid identification of hazardous materials is required (e.g., [34]).

## Methods

### *Bacillus *spores

*Bacillus *type strains were purchased from German Collection of Microorganisms and Cell Cultures (DSMZ): *Bacillus atrophaeus *DSMZ 7264, *Bacillus brevis *DSMZ 30 (reclassified as *Brevibacillus brevis *[24]), *Bacillus circulans *DSMZ 11, *Bacillus lentus *DSMZ 9, *Bacillus thuringiensis *DSMZ 2046. *Bacillus subtilis *(clone BD 170) was obtained from bio-protect (Gesellschaft für biologischen Pflanzenschutz, Konstanz, Germany). *Bacillus megaterium *was from our own culture collection. All type strains belong to the RNA group 1, except *Brevibacillus brevis*, which belongs to RNA group 4. All bacilli were cultured in liquid Medium 1 suggested by DSMZ consisting of (in g/l) peptone (5.0) and meat extract (3.0), with a prolonged incubation of 10 to 15 days at 150 rpm and 30°C to deplete the medium. To initiate and force sporulation, cultures were centrifuged under sterile conditions and the pellets were transferred to a sporulation medium with the following composition (in g/l): NH_4_Cl (1.6); yeast extract (1.0); K_2_HPO_4 _(0.9); KH_2_PO_4 _(0.6); MgSO_4 _• 7H_2_O (0.2); CaCl_2 _• 2H_2_O (0.07); FeSO_4 _• 7H_2_O (0.01); EDTA (0.01); trace element solution (1 ml/l) containing MnSO_4 _• H_2_O (0.02); (NH_4_)_6_Mo_7_O_24 _• 4H_2_O (0.02); H_3_BO_4 _(0.01); CuSO_4 _• 5H_2_O (0.01); ZnSO_4 _• 7H2O (0.01). After an additional incubation period of approximatively 30 days, cultures were checked microscopically and spores were harvested by centrifugation, washed three times with distilled water to remove media compounds and subsequently freeze dried. Bentonite was ground to a fine powder using a ball mill. Spores of *B. megaterium *were mixed with powdered bentonite in different wt/wt ratios (1:1; 1:2; 1:4; 1:6: 1:8) and immediately measured by FTIR without any further treatment. To convert wt/wt ratios to colony forming units (cfu) per gram of mineral matrix, a 3.1 mg aliquot of the 1:8 mixture was suspended in 10 mg of sterile tap water, sonicated for 1 min, and boiled for 5 min to kill vegetative cells of contaminating microorganisms. The suspension was serially diluted and and 20 μl aliquots were plated in triplicates onto a growth medium (HiCrome' Bacillus Agar, Sigma-Aldrich, Buchs, Switzerland) highly selective for *Bacillus *species [35]. Plates were incubated at 30°C for 48 h and colonies counted by visual inspection.

### Fourier transform infrared spectroscopy

A JASCO 4200-FTIR (Brechbühler AG, Schlieren, Switzerland) was used for the measurements in attenuated total reflection (ATR) mode using an ATR accessory equipped with a zinc selenide (ZnSe) prism. A small amount (approximately 2 to 5 mg) of sample - enough to cover the prism - was placed onto the ATR accessory and spectra were collected. Fifty scans with a resolution of 4 cm^-1 ^using the ZnSe prism were averaged and used for further chemometrical analyses. For measurements in the transmission mode, samples were suspended in ultra pure water (18.2 Ω) and 100 μl were pipetted onto a thin polyethylene (PE) film held in place by in a slide mount and air dried. Spectra were collected with the same settings as with the ZnSe prism. Pure PE film was taken as background. Measurement range was 650 to 4000 cm^-1 ^for ATR and 400 to 4000 cm^-1 ^for transmission mode. Each sample was measured in three to five independent replicates.

### Chemometrical data processing

Raw spectral data were processed with JASCO Spectra Manager 2.02.02. First, all spectra were treated as follows: (i) baseline correction (linear); (ii) ATR-correction (for reflection mode only); (iii) smoothing (Savitzky-Golay, width = 15); (iv) truncate (1800 - 650 cm^-1 ^for ATR mode, none for transmission mode); (v) normalization (hightest value = 1, lowest value = 0). Subsequently, the second derivative was calculated (Savitzky-Golay, width = 5 for ATR mode, 15 for transmission mode). Second derivatives of corrected spectra were compared applying hierarchical cluster analysis, HCA, applying Ward's minimum variance method and squared Euclidian distance, as well as principal components analysis, PCA. Statistical analyses were performed with either the open source software package R or SPSS 12.0.1.

## Authors' contributions

Main experimental work was carried out by ABA as part of her PhD thesis under the supervision of HB. HB was the principal investigator and contributed also experimental work and statistical analyses. Both ABA and HB wrote the manuscript. Both authors read and approved the final manuscript.

## Acknowledgements

The work was supported by the Biosafety Office of the Canton of Zurich (Zurich, Switzerland). We thank Beat Köhler (Brechbühler AG, Schlieren, Switzerland) for his help and advice regarding the implementation of infrared spectroscopy in our lab. The help of Dr. Timothy Paine during the revision of the manuscript is highly appreciated.
